# Socioeconomic Disparities in eHealth Literacy and Preventive Behaviors During the COVID-19 Pandemic in Hong Kong: Cross-sectional Study

**DOI:** 10.2196/24577

**Published:** 2021-04-14

**Authors:** Ziqiu Guo, Sheng Zhi Zhao, Ningyuan Guo, Yongda Wu, Xue Weng, Janet Yuen-Ha Wong, Tai Hing Lam, Man Ping Wang

**Affiliations:** 1 School of Nursing University of Hong Kong Hong Kong China (Hong Kong); 2 School of Public Health University of Hong Kong Hong Kong China (Hong Kong)

**Keywords:** COVID-19, eHealth literacy, preventive behaviors, socioeconomic disparities, web-based information seeking

## Abstract

**Background:**

eHealth literacy can potentially facilitate web-based information seeking and taking informed measures.

**Objective:**

This study aimed to evaluate socioeconomic disparities in eHealth literacy and seeking of web-based information on COVID-19, and their associations with COVID-19 preventive behaviors.

**Methods:**

The COVID-19 Health Information Survey (CoVHIns), using telephonic (n=500) and web-based surveys (n=1001), was conducted among adults in Hong Kong in April 2020. The Chinese eHealth literacy scale (eHEALS; score range 8-40) was used to measure eHealth literacy. COVID-19 preventive behaviors included wearing surgical masks, wearing fabric masks, washing hands, social distancing, and adding water or bleach to the household drainage system. Adjusted beta coefficients and the slope indices of inequality for the eHEALS score by socioeconomic status, adjusted odds ratios (aORs) for seeking of web-based information on COVID-19 by socioeconomic status, and aORs for the high adherence to preventive behaviors by the eHEALS score and seeking of web-based information on COVID-19 were calculated.

**Results:**

The mean eHEALS score was 26.10 (SD 7.70). Age was inversely associated with the eHEALS score, but education and personal income were positively associated with the eHEALS score and seeking of web-based information on COVID-19 (for all, *P* for trend<.05). Participants who sought web-based information on COVID-19 showed high adherence to the practice of wearing surgical masks (aOR 1.56, 95% CI 1.15-2.13), washing hands (aOR 1.33, 95% CI 1.05-1.71), social distancing (aOR 1.48, 95% CI 1.14-1.93), and adding water or bleach to the household drainage system (aOR 1.67, 95% CI 1.28-2.18). Those with the highest eHEALS score displayed high adherence to the practice of wearing surgical masks (aOR 3.84, 95% CI 1.63-9.05), washing hands (aOR 4.14, 95% CI 2.46-6.96), social distancing (aOR 2.25, 95% CI 1.39-3.65), and adding water or bleach to the household drainage system (aOR 1.94, 95% CI 1.19-3.16), compared to those with the lowest eHEALS score.

**Conclusions:**

Chinese adults with a higher socioeconomic status had higher eHealth literacy and sought more web-based information on COVID-19; both these factors were associated with a high adherence to the guidelines for preventive behaviors during the COVID-19 pandemic.

## Introduction

Curbing of the spread of COVID-19 depends on the timely adoption of appropriate preventive behaviors by the public. Web-based health information is important in affecting preventive behaviors, particularly when physical distancing and stay-at-home orders during the pandemic have reduced face-to-face health communication [1]. A recent study reported that seeking of web-based information on COVID-19 from social networking apps and internet-based news media are associated with preventive behaviors [2]. A breadth of information and misinformation has been disseminated on the internet and rapidly propagated and evolved on social media platforms [3]. Exposure to misinformation on the internet or conspiracy theories regarding COVID-19 are associated with decreased adherence to prevention guidelines and worse physical and mental health outcomes [4,5]. The ability to seek, understand, and appraise health information on the internet and ultimately take well-informed action to handle health problems can be assessed on the basis of eHealth literacy [6]. A higher eHealth literacy is associated with more active information seeking and scrutiny [7,8]. Lack of access or capacity to understand health information on the internet, in contrast, is associated with negligence toward health warnings and difficulty in making health decisions [9].

Appropriate processing and utilization of health information is complex during the COVID-19 pandemic, given the novel outbreak patterns and evolving information regarding the disease [10]. It is important to identify the characteristics of individuals at the risk of lower eHealth literacy for effective health promotion, including the provision of limited literacy resources [11]. Previous studies have suggested that eHealth literacy is affected by sociodemographic, environmental, and contextual factors [12]. Disparities in eHealth literacy by education and income have been previously reported [13], but incongruent correlations between socioeconomic status and eHealth literacy have been found across populations with different characteristics [8,14,15]. The COVID-19 pandemic has disproportionately affected the lower socioeconomic status (SES) group and has limited access to health care, overcrowded living conditions with a higher risk of disease transmission, and inconvenienced individuals who are in occupations that do not allow working from home [16], which have further accentuated existing socioeconomic inequalities. Since eHealth literacy skill is not static and evolves with changes in new social contexts [6], little is known about the disparities in eHealth literacy in the unique context of widening socioeconomic inequalities and the overwhelming influx of COVID-19–related information (and misinformation) being disseminated.

Hong Kong, the most developed and westernized city of China, has a larger income gap (Gini index 0.539 in 2016) compared to other developed countries [17], but internet use is prevalent across individuals of different SES because of the advanced cyber infrastructure and the low cost of internet access [18]. Nearly all individuals have sought web-based information during the COVID-19 pandemic [19]. Our previous study in 2009-2012 reported that disparities in SES groups affected web-based health information seeking behavior [20]. Considering that the context of COVID-19 may stimulate universal web-based information seeking behavior by triggering effective responses such as fear and anxiety [21], it remains unknown whether SES disparities in web-based health information seeking existed during the COVID-19 pandemic. Our study’s research questions were as follows: (1) Are there socioeconomic disparities in seeking web-based information on COVID-19 during the pandemic? (2) Are there socioeconomic disparities in eHealth literacy among these web-based information seekers? (3) Is seeking of web-based information and eHealth literacy associated with preventive behaviors during the COVID-19 pandemic? In a random cohort of adults in Hong Kong, we examined socioeconomic disparities in seeking web-based information on COVID-19 and eHealth literacy, and their associations with personal preventive behaviors during the COVID-19 pandemic.

## Methods

### Design and Participants

This study was part of the COVID-19 Health Information Survey (CoVHIns)—which is a cross-sectional survey among adults in Hong Kong who are aged ≥18 years—which investigated COVID-19–related information use, preventive behaviors, and well-being. The survey was conducted on April 9-23, 2020, after the peak of the second wave of the outbreak, and when social distancing measures were implemented. Data were collected using telephonic and web-based surveys. All interviews were conducted by trained interviewers of Social Policy Research Limited through the Web-based Computer Assisted Telephone Interview system.

The details of CoVHIns have been reported previously [22,23]. Briefly, a 2-stage sampling method was adopted for the telephonic survey. First, telephone numbers were retrieved from the residential telephone directories and randomly listed for interview. Invalid numbers, lack of responses (after being called for a maximum of 5 times), and ineligible households (including individuals aged <18 years or those who are unable to communicate in Cantonese or Mandarin) were excluded. Second, once a household was successfully contacted, the eligible family member whose birth date was closest to the interview date was invited to complete the interview. Each interview lasted approximately 20 minutes. A total of 816 landline telephone numbers were successfully sampled, and 500 participants consented to and completed the interview (response rate=61.3%).

In addition, web-based surveys randomly sampled participants from a representative panel of >100,000 mobile phone users, which was generated by sending text messages to a random list of mobile phone numbers provided by the Numbering Plan for Telecommunication Services (prefixes 5, 6, 9). Stratified random sampling by sex and age was adopted. Text messages with an invitation were sent to the randomly selected members in the panel. Among 1623 eligible individuals contacted, 1001 participants consented to and completed the questionnaire on the internet (response rate=61.7%). Ethics approval was granted by the Institutional Review Board of the University of Hong Kong/Hospital Authority Hong Kong West Cluster (approval# UW-20-238).

### Measurements

Seeking of web-based information on COVID-19 was self-reported (sought or no-sought). eHealth literacy was assessed among individuals who had sought web-based information on COVID-19, considering that eHealth literacy is based on the experience of access to web-based information [24]. We used the Chinese version of the eHealth literacy scale (eHEALS) to measure eHealth literacy levels by asking participants’ about their past experience in seeking web-based information on COVID-19 (Multimedia Appendix 1). The eHEALS contains 8 items scored with a 5-point Likert scale (ranging from “strongly disagree” to “strongly agree”). The total score ranged from 8 to 40, with a higher score indicating higher eHealth literacy [25]. A Cronbach *α* of .95 was used in our study. Consistent with the Chinese version of eHEALS [25], we observed a unidimensional structure of the Chinese eHEALS with adequate model fitness (comparative fit index 0.974 [>0.95, acceptable], root mean square error of approximation 0.097 [close to 0.06, acceptable], and Tucker-Lewis index 0.964 [>0.95 acceptable]) [26]. We divided the eHEALS score into 4 categories (Q1-Q4) based on quartile values (median 28, IQR 22-32) in accordance with previous studies, using the median as the cutoff [7,27]. Specifically, Q1 was the interval of the overall eHEALS score ranging from the lowest value (ie, 8) to a score of 22; Q2, from 22 to 28; Q3, from 28 to 32; and Q4, from 32 to the highest value (ie, 40).

Based on World Health Organization guidelines for COVID-19 prevention [28], we assessed personal preventive behaviors in the past 7 days, including the following: “wearing surgical masks when going out,” “wearing fabric masks when going out,” “washing hands with alcohol-based sanitizer,” “adding water/bleach to the household drainage system,” and “keeping a social distance from people in public areas (eg, 1.5 meters),” with responses of “never,” “occasionally,” “sometimes,” and “often” (Multimedia Appendix 2). Adherence to personal preventive behavior was dichotomized (low or high adherence) on the basis of previous studies on the association between eHealth literacy and health behaviors [14,24]. Responses of “never,” “occasionally,” and “sometimes” were together considered as low adherence and “often” was considered as high adherence.

Education levels and income were considered as indicators of SES. Education levels were measured as categorical variables (“primary or below,” “secondary,” and “tertiary or above”) on the basis of the highest education level attained. We measured monthly personal income in accordance with 6 predefined categories (from ≤HK $10,000-$50,001 [US $1=HK $7.8]). Since few participants had an income of HK $40,001-$50,000 and ≥HK $50,001, the data were recoded in 4 categories including ≤HK $10,000, HK $10,001-$20,000, HK $20,001-$30,000, and >HK $30,000 to obtain robust outcomes on regression analyses.

Other demographic data included sex, age, and marital status (never been married, married or cohabitating, and divorced or separated or widowed). Employment status was categorized as economically active (full-time work or part-time work) and economically inactive (student, homemaker, unemployed, and retiree) [29]. Any chronic diseases were self-reported (any or none).

### Statistical Analysis

All data were weighted by sex, age, and education levels in accordance with the 2016 population by census to improve the representativeness of the sample.

First, disparities in seeking web-based information on COVID-19 (dichotomized variable) by SES were assessed through multivariable logistic regression, which yielded adjusted odds ratios (aOR) for seeking web-based information on COVID-19. Second, socioeconomic disparities in eHealth literacy, being a continuous variable, were assessed through linear regression, which yielded unstandardized regression coefficients to reflect the change in the eHEALS score for a unit change in the independent variable. Third, we used the slope index of inequality (SII) to estimate the absolute difference in the eHEALS score between the most advantaged and most disadvantaged groups. The SII has been recommended by the World Health Organization, and a high SII indicates severe inequality [30]. Income categories were first ranked from the lowest to highest, and the cumulative proportion of participants were assigned to each category on the basis of the midpoint of range as the code for each category. The eHEALS score was then regressed against the cumulative proportion of each income category [30]. A similar analysis was performed for education-related SII. As each personal preventive behavior was dichotomized as low and high adherence, the associations (determined with aOR and 95% CI values) of seeking web-based information on COVID-19 and the eHEALS score with each personal preventive behavior were estimated through multivariable logistic regression adjusted for demographic variables, SES, and chronic disease. All analyses were performed using Stata (version 15.1, Stata Corp).

## Results

Table 1 shows the weighted sample (N=1501; females: n=829, 52.6%), with 495 (27.7%) participants aged ≥60 years. Approximately two-third (66.1%) participants were married or cohabitating, and 981 (62.9%) were economically active. Most participants had attained secondary or tertiary and above education. In total, 519 (37.5%) participants’ monthly personal income was HK ≤$10,000, and 1040 (67.8%) participants self-reported seeking web-based information on COVID-19. The mean eHEALS score was 26.10 (SD 7.70).

**Table 1:** Demographic characteristics, socioeconomic status, chronic disease outcomes, and seeking of web-based information on COVID-19 among the study participants (N=1501).

Characteristics		Participants		
		Number	Unweighted %	Weighted %^a^
**Sex**				
	Male	672	44.8	47.5
	Female	829	55.2	52.6
**Age (years)**				
	18-39	497	33.1	33.8
	40-59	509	33.9	38.5
	≥60	495	33.0	27.7
**Marital status**				
	Never been married	353	23.5	24.7
	Married/cohabitating	1053	70.2	66.1
	Divorced/separated/widowed	95	6.3	9.2
**Education**				
	Primary or below	247	16.5	23.2
	Secondary	864	57.6	45.4
	Tertiary or above	390	26.0	31.4
**Income (HK $)^b^**				
	≤10,000	519	34.6	37.5
	10,001-20,000	519	34.6	30.7
	20,001-30,000	268	17.9	17.5
	>30,000	195	13.0	14.3
**Employment status**				
	Economically active	981	65.4	62.9
	Economically inactive	520	34.6	37.1
**Chronic diseases^c^**				
	Any	187	12.5	15.0
	None	1314	87.5	85.0
**Seeking web-based information on COVID-19**				
	Yes	1040	69.3	67.8
	No	461	30.7	32.2

^a^Weighted by sex, age, and education levels in accordance with the 2016 population by census.

^b^US $1=HK $7.8.

^c^Participants self-reported being diagnosed with any chronic disease (eg, hypertension, diabetes, or cancer).

Table 2 shows the inverse correlation between age and seeking of web-based information on COVID-19 (*P* for trend<.001). Education (secondary education: aOR 1.55, 95% CI 1.10-2.18; tertiary or above education: aOR 2.98, 95% CI 1.84-4.81; *P* for trend<.001), income (*P* for trend=.025), and the absence of chronic diseases (aOR 1.56, 95% 1.11-2.21) were associated with seeking of web-based information on COVID-19.

**Table 2:** Associations among demographic variables, socioeconomic status, and chronic disease with seeking of web-based information on COVID-19 (N=1501).

Characteristics			Sought (n=1040), n (%)^a^		No-sought (n=461), n (%)^a^		Association		
							Unadjusted OR^b^ (95% CI)		Adjusted OR^c^ (95% CI)
**Sex**									
	Male	481 (49.6)		191 (42.9)		1		1	
	Female	559 (50.4)		270 (57.1)		0.82 (0.66-1.03)		0.83 (0.65-1.06)	
**Age (years)**									
	18-39	411 (41.4)		86 (17.8)		1		1	
	40-59	391 (41.4)		118 (32.4)		0.69 (0.51-0.95)^d^		0.86 (0.60-1.23)	
	≥60	238 (17.1)		257 (49.9)		0.19 (0.14-0.26)^e^		0.40 (0.27-0.61)^e^	
**Marital status**									
	Never been married	290 (30.1)		63 (13.5)		1		1	
	Married/cohabitating	702 (64.4)		351 (69.7)		0.43 (0.32-0.59)^e^		0.90 (0.62-1.30)	
	Divorced/separated/widowed	48 (5.5)		47 (16.8)		0.22 (0.14-0.36)^e^		0.65 (0.37-1.15)	
**Education**									
	Primary or below	105 (13.2)		142 (44.3)		1		1	
	Secondary	597 (47.1)		267 (41.8)		3.02 (2.26-4.04)^e^		1.55 (1.10-2.18)^d^	
	Tertiary or above	338 (39.8)		52 (13.9)		8.79 (5.98-12.93)^e^		2.98 (1.84-4.81)^e^	
**Income (HK $)^f^**									
	≤10,000	304 (28.7)		215 (55.9)		1		1	
	10,001-20,000	360 (32.3)		159 (27.4)		1.60 (1.24-2.07)^e^		0.97 (0.69-1.36)	
	20,001-30,000	208 (20.6)		60 (11.0)		2.45 (1.75-3.43)^e^		1.06 (0.69-1.63)	
	>30,000	168 (18.4)		27 (5.7)		4.40 (2.83-6.85)^e^		1.79 (1.04-3.06)^d^	
**Employment status**									
	Economically inactive	293 (27.5)		227 (57.4)		1		1	
	Economically active	747 (72.5)		234 (42.6)		2.47 (1.97-3.10)^e^		1.18 (0.84-1.65)	
**Chronic diseases^g^**									
	Any	91 (9.5)		96 (26.7)		1		1	
	None	949 (90.5)		365 (73.3)		2.74 (2.01-3.74)^e^		1.56 (1.11-2.21)^d^	

^a^The proportion weighted by sex, age, and education levels in accordance with the 2016 population by census.

^b^OR: odds ratio

^c^Mutually adjusted for the variables in the table.

^d^*P*<.05.

^e^*P*<.001.

^f^US $1=HK $7.8.

^g^Self-reported by participants if having been diagnosed with a chronic disease (eg, hypertension, diabetes, or cancer).

Table 3 shows the inverse correlation between age and the eHEALS score (*P* for trend<.001). Education (secondary education: adjusted *β* 3.58, 95% CI 1.98-5.18; tertiary or above education: adjusted *β* 6.22, 95% CI 4.39-8.06; *P* for trend<.001) and income (*P* for trend<.001) were associated with the eHEALS score. The estimated difference in the eHEALS score between participants of the highest and the lowest SES was higher by education than by income (SII 13.27 vs 7.30). Sex, marital status, employment status, and chronic diseases were not associated with the eHEALS score after adjusting for age and SES.

**Table 3:** Associations among demographic variables, socioeconomic status, and chronic diseases with the eHealth literacy score^a^ among participants seeking web-based information on COVID-19 (N=1040).

Characteristics		Mean (SD)	Unadjusted *β* (95% CI)	Adjusted *β* (95% CI)^b^	SII^c^
**Sex**					N/A^d^
	Male	26.00 (7.51)	0	0	
	Female	26.19 (7.86)	0.19 (–0.75 to 1.13)	–0.01 (–0.83 to 0.80)	
**Age (years)**					N/A
	18-39	28.84 (6.07)	0	0	
	40-59	27.18 (6.12)	–1.67 (–2.61 to –0.72)^e^	–0.77 (–1.82 to 0.28)	
	≥60	19.60 (8.79)	–9.24 (–10.33 to –8.16)^f^	–5.48 (–6.91 to –4.05)^f^	
**Marital status**					N/A
	Never been married	28.99 (5.93)	0	0	
	Married/cohabitating	25.07 (7.93)	–3.92 (–4.95 to –2.90)^f^	–1.03 (–2.09 to 0.02)	
	Divorced/separated/widowed	23.65 (9.07)	–5.35 (–7.64 to –3.06)^f^	–1.83 (–3.93 to 0.27)	
**Education**					13.27^f^
	Primary or below	17.56 (8.45)	0	0	
	Secondary	25.40 (6.80)	7.84 (6.42 to 9.26)^f^	3.58 (1.98 to 5.18)^f^	
	Tertiary or above	29.98 (6.34)	12.42 (10.92 to 13.92)^f^	6.22 (4.39 to 8.06)^f^	
**Income (HK $)^g^**					7.30^f^
	≤10,000	23.86 (8.27)	0	0	
	10,001-20,000	25.38 (7.63)	1.52 (0.38 to 2.65)^e^	–0.40 (–1.69 to 0.88)	
	20,001-30,000	27.74 (6.74)	3.88 (2.57 to 5.19)^f^	0.62 (–0.86 to 2.10)	
	>30,000	29.67 (6.07)	5.81 (4.41 to 7.22)^f^	2.25 (0.63 to 3.88)^e^	
**Employment status**					N/A
	Economically inactive	23.40 (8.75)	0	0	
	Economically active	27.16 (6.97)	3.76 (2.74 to 4.78)^f^	0.39 (–0.89 to 1.66)	
**Chronic diseases**					N/A
	None	26.42 (7.50)	0	0	
	Any	22.71 (8.89)	–3.71 (–5.35 to –2.07)^f^	–0.85 (–2.31 to 0.60)	

^a^eHealth literacy scores ranged between 8 and 40, with higher scores indicating higher eHealth literacy.

^b^Mutually adjusted for the variables in the table.

^c^SII: slope index of inequality; SII refers to the absolute difference in the eHEALS score between the most advantaged and most disadvantaged groups, a higher score indicating a higher disparity in the eHEALS score.

^d^N/A: not applicable.

^e^*P*<.01.

^f^*P*<.001.

^g^US $1=HK $7.8.

Table 4 shows that participants who had sought web-based information on COVID-19 displayed higher adherence to the practice of wearing surgical masks (aOR 1.56, 95% CI 1.15-2.13), washing hands with alcohol-based sanitizers (aOR 1.33, 95% CI 1.05-1.71), adding water or bleach to the household drainage system (aOR 1.67, 95% CI 1.28-2.18), and social distancing (aOR 1.48, 95% CI 1.14-1.93) than those who did not seek web-based information on COVID-19. Seeking of web-based information on COVID-19 was not associated with the adherence to the practice of wearing a fabric mask. Among those who sought web-based information on COVID-19, the eHEALS score was associated with the adherence to the practice of wearing surgical masks (Q2: aOR 1.44, 95% CI 0.91-2.30; Q3: aOR 2.05, 95% CI 1.26-3.35; Q4: aOR 3.84, 95% CI 1.63-9.05; *P* for trend<.001; overall score: aOR 1.04, 95% CI 1.01-1.07). Regarding the adherence to washing hands with alcohol-based sanitizers, the aOR was 1.77 (95% CI 1.25-2.53) for Q2, 2.16 (95% CI 1.52-3.09) for Q3, 4.14 (95% CI 2.46-6.96) for Q4 (*P* for trend<.001), and 1.06 (95% CI 1.04-1.08) for the overall eHEALS score. Similarly, the eHEALS score was associated with the adherence to adding water or bleach to the household drainage system (Q2: aOR 1.47, 95% CI 1.02-2.15; Q3: aOR 1.89, 95% CI 1.30-2.75; Q4: aOR 1.94, 95% CI 1.19-3.16; *P* for trend=.001; overall score: aOR 1.04, 95% CI 1.02-1.06) and social distancing (Q2: aOR 1.68, 95% CI 1.16-2.44; Q3: aOR 1.58, 95% CI 1.09-2.30; Q4: aOR 2.25, 95% CI 1.39-3.65; *P* for trend=.002; overall score: aOR 1.03, 95% CI 1.01-1.05). We observed no association between the eHEALS score and the practice of wearing fabric masks.

**Table 4:** Association between the adherence to preventive behaviors by seeking web-based information on COVID-19 and the eHealth literacy score.

Parameter	Wearing a surgical mask^a^					Wearing a fabric mask^a^						Washing hands with alcohol-based sanitizers^a^						Adding water or bleach to the household drainage system^a^						Social distancing (eg, by 1.5 meters)^a^			
	n (%)	aOR^b^ (95% CI)	*P* for trend value		n (%)		aOR (95% CI)	*P* for trend value		n (%)			aOR (95% CI)	*P* for trend value		n (%)			aOR (95% CI)	*P* for trend value		n (%)			aOR (95% CI)	*P* for trend value	
**Sought web-based information on COVID-19 (n=1501)**				N/A^c^					N/A						N/A						N/A						N/A
No	359 (77.9)	1			85 (18.4)		1			191 (41.4)			1			122 (26.5)			1			122 (26.5)			1		
Yes	899 (86.4)	1.56 (1.15-2.13)^d^			166 (16.0)		0.84 (0.61-1.15)			572 (55.0)			1.33 (1.05-1.71)^e^			385 (37.0)			1.67 (1.28-2.18)^f^			377 (36.3)			1.48 (1.14-1.93)^d^		
**eHealth literacy score categories for seekers of web-based information on COVID-19 (n=1040)**				<.001					.39						<.001						.001						.002
Q1^g^	224 (79.4)	1			38 (13.5)		1			109 (38.7)			1			83 (29.4)			1			76 (27.0)			1		
Q2	244 (85.9)	1.44 (0.91-2.30)			44 (15.5)		1.17 (0.72-1.90)			153 (53.9)			1.77 (1.25-2.53)^d^			102 (35.9)			1.47 (1.02-2.15)^e^			108 (38.0)			1.68 (1.16-2.44)^d^		
Q3	309 (89.6)	2.05 (1.26-3.35)^d^			64 (18.6)		1.39 (0.86-2.22)			210 (60.9)			2.16 (1.52-3.09)^f^			142 (41.2)			1.89 (1.30-2.75)^d^			130 (37.7)			1.58 (1.09-2.30)^e^		
Q4	122 (94.6)	3.84 (1.63-9.05)^d^			20 (15.5)		1.12 (0.59-2.12)			100 (77.5)			4.14 (2.46-6.96)^f^			58 (45.0)			1.94 (1.19-3.16)^d^			63 (48.8)			2.25 (1.39-3.65)^d^		
**eHealth literacy score (continuous variable) for seekers of web-based information on COVID-19 (n=1040)**				N/A					N/A						N/A						N/A						N/A
Overall score		1.04 (1.01-1.07)^d^					1.01 (0.99-1.04)						1.06 (1.04-1.08)^f^						1.04 (1.02-1.06)^f^						1.03 (1.01-1.05)^d^		

^a^All preventive behaviors: high adherence (“often”) vs low adherence (“never,” “occasionally,” and “sometimes”).

^b^aOR: adjusted odds ratio; the aOR was adjusted for sex, age, marital status, employment, education, income, and chronic diseases.

^c^N/A: not applicable.

^d^*P*<.01.

^e^*P*<.05.

^f^*P*<.001.

^g^The eHealth literacy score was divided into 4 categories (Q1-Q4) on the basis of the quartile values (median 28, IQR 22-32); a higher score indicated higher eHealth literacy.

## Discussion

### Principal Findings

This study is the first to report socioeconomic disparities in seeking web-based information on COVID-19 and eHealth literacy during the COVID-19 pandemic and their association with a high adherence to COVID-19–related preventive behaviors, including wearing surgical masks, washing hands, adding water or bleach to the household drainage system, and social distancing. 

Seeking of web-based information on COVID-19 was observed among younger participants in our study, concurrent with previous studies on web-based health information seeking behaviors [31]. A recent study also indicated that younger family members sought web-based information for the elderly during the pandemic [32]. Such an age disparity in information seeking can be attributed to the higher penetration rate of internet devices such as personal computers and smartphones among younger rather than older individuals [18]. Small font sizes, crowded visual presentations, and distracting flashes on most web-based information sources could be barriers to web-based information seeking among the elderly [33]. More frequent health information seeking from traditional media such as the radio and newspapers were observed among the elderly in our previous population-based study [20]. Our finding that higher SES including education levels and income is associated with seeking web-based information on COVID-19 is consistent with that of previous studies on seeking of web-based health information conducted before the COVID-19 pandemic [13,31]. Compared to our previous study, which measured SES disparities in seeking of web-based health information in 2009-2012, the ORs of web-based information seeking were found to decrease (eg, tertiary or above education: 2.98 in 2020 vs 8.00 in 2009-2012) [20]. Such a reduction in the effect size could result from the increased popularity of internet-accessible devices in the general population in Hong Kong [34]. Alternatively, the decreased ORs could be attributed to increased information seeking behaviors in crisis events, which were suggested as a means of reducing situation uncertainty and controlling risk [35].

Furthermore, we found age and SES disparities in eHealth literacy, thus revealing disparities in locating, understanding, and the utility of web-based information among those who sought web-based information on COVID-19. The associations between sociodemographic characteristics (eg, age and SES) and eHealth literacy observed in our study were concurrent with previous findings on health literacy [36,37]. Our study focused on eHealth literacy because the Internet has been the major platform for disseminating health information during the COVID-19 pandemic, since it is easily available and can instantly update information on, for example, preventive behaviors and access to social and health services. Considering that web-based information is complex and misinformation on the internet has led to inappropriate behaviors [38], those who used the internet for health information but with limited eHealth literacy skills to discern the quality of different information sources were the potential at-risk populations and require more attention. We observed stronger associations between eHealth literacy and education rather than income, which probably reflects the notion that knowledge and skills are more affected by cognitive function than by the available material. The education-related disparity in eHealth literacy was larger than the income-related disparity in our study, which suggest that education plays a more crucial role than income in affecting eHealth literacy. Other studies have also suggested that disparities in eHealth literacy were due to knowledge gaps rather than merely physical barriers to internet access [39]. Furthermore, we noticed that eHealth literacy and seeking of web-based information on COVID-19 have similar risk factors including an older age and a lower SES [13]. eHealth literacy enables seeking of web-based information [8]; further studies can explore the extent to which low eHealth literacy hinders seeking of web-based information among older and low-SES individuals.

Successful control of the COVID-19 pandemic would need universal adherence to preventive behaviors, which have proven very effective in reducing disease spread [40]. Seeking of web-based information on COVID-19 is associated with the adherence to preventive behaviors, suggesting the need to understand the barriers to the low SES group, including low eHealth literacy to use the internet to obtain health information. Our participants with higher eHealth literacy showed high adherence to personal preventive behaviors, which was consistent with the results of previous non–COVID-19 studies that eHealth literacy correlated with health behaviors such as regular physical exercise and balanced diets [24,41,42]. Our study extended those findings to COVID-19 preventive behaviors in the specific context of the COVID-19 pandemic, in which increasing misinformation has been disseminated through the internet. Low eHealth literacy could lead to difficulties in fact-checking and mistrust in peoples’ beliefs in coronavirus conspiracies, which would impede the performance of preventive behaviors [43]. Such disparities in eHealth literacy have led to disparities in guidelines on preventive behaviors, their profound consequence being health inequality [44]. Web-based information should be better designed to address the eHealth literacy levels of target users, particularly those with a low SES, to bridge the existing knowledge gap. Further studies are needed to explore how to improve eHealth literacy effectively and the approach involving the use of eHealth literacy to facilitate better health behaviors. 

### Limitations

Our study has some limitations. First, the cross-sectional data cannot confirm the causal association, although it is unlikely that higher eHealth literacy or seeking of web-based information on COVID-19 would lead to higher education and income. Second, we measured the perceived eHealth literacy instead of actual performance on the internet. Some studies have measured performed eHealth literacy and reported a weak or moderate correlation between perceived and performed eHealth literacy [15,45]. Third, eHEALS, the most commonly used validated scale, was developed at the early stage of internet technology; its fit with Web 2.0-related technologies (social media) was not clear because of the considerable changes on the internet (more participatory and interactive web) [46]. Future studies are needed to improve the model of eHealth literacy with the evolution of the internet and the COVID-19 pandemic [46,47]. Fourth, we did not collect data on channels of web-based information on COVID-19; hence, further studies should include details of the frequency and channels on the internet for seeking information on COVID-19.

### Conclusions

This study provides the first evidence that Chinese adults with a higher SES had higher eHealth literacy and sought web-based information on COVID-19, and that both these factors are associated with high adherence to the guidelines on preventive behaviors during the COVID-19 pandemic. Effective interventions are needed to enhance the low eHealth literacy skills of low-SES individuals to combat the COVID-19 pandemic.

## Appendix

Multimedia Appendix 1 eHealth Literacy Scale.

Multimedia Appendix 2 Unweighted prevalence of preventive behaviors by online COVID-19 information seeking (N=1501).

## Abbreviations

aOR: adjusted odds ratio
eHEALS: eHealth literacy scale
SES: socioeconomic status
SII: slope indices of inequality

## References

[1] Zarocostas J (2020). How to fight an infodemic. Lancet.

[2] Liu PL (2020). COVID-19 Information Seeking on Digital Media and Preventive Behaviors: The Mediation Role of Worry. Cyberpsychol Behav Soc Netw.

[3] Xie B, He D, Mercer T, Wang Y, Wu D, Fleischmann K, Zhang Y, Yoder L, Stephens K, Mackert M, Lee M (2020). Global health crises are also information crises: A call to action. J Assoc Inf Sci Technol.

[4] Tasnim S, Hossain MM, Mazumder H (2020). Impact of Rumors and Misinformation on COVID-19 in Social Media. J Prev Med Public Health.

[5] Freeman D, Waite F, Rosebrock L, Petit A, Causier C, East A, Jenner L, Teale A, Carr L, Mulhall S, Bold E, Lambe S (2020). Coronavirus conspiracy beliefs, mistrust, and compliance with government guidelines in England. Psychol Med.

[6] Norman CD, Skinner HA (2006). eHealth Literacy: Essential Skills for Consumer Health in a Networked World. J Med Internet Res.

[7] Neter E, Brainin E (2012). eHealth literacy: extending the digital divide to the realm of health information. J Med Internet Res.

[8] Wong DK, Cheung M (2019). Online Health Information Seeking and eHealth Literacy Among Patients Attending a Primary Care Clinic in Hong Kong: A Cross-Sectional Survey. J Med Internet Res.

[9] Pirisi A (2000). Low health literacy prevents equal access to care. Lancet.

[10] Beaunoyer E, Dupéré S, Guitton MJ (2020). COVID-19 and digital inequalities: Reciprocal impacts and mitigation strategies. Comput Human Behav.

[11] Morgan-Daniel J, Ansell M, Adkins Le (2020). COVID-19 Patient Education and Consumer Health Information Resources and Services. J Consum Health Internet.

[12] Levin-Zamir D, Bertschi I (2018). Media Health Literacy, eHealth Literacy, and the Role of the Social Environment in Context. Int J Environ Res Public Health.

[13] Tennant B, Stellefson M, Dodd V, Chaney B, Chaney D, Paige S, Alber J (2015). eHealth literacy and Web 2.0 health information seeking behaviors among baby boomers and older adults. J Med Internet Res.

[14] Mitsutake S, Shibata A, Ishii K, Oka K (2012). Association of eHealth literacy with colorectal cancer knowledge and screening practice among internet users in Japan. J Med Internet Res.

[15] van der Vaart R, van Deursen AJ, Drossaert CH, Taal E, van Dijk JA, van de Laar MA (2011). Does the eHealth Literacy Scale (eHEALS) measure what it intends to measure? Validation of a Dutch version of the eHEALS in two adult populations. J Med Internet Res.

[16] Patel JA, Nielsen FBH, Badiani AA, Assi S, Unadkat VA, Patel B, Ravindrane R, Wardle H (2020). Poverty, inequality and COVID-19: the forgotten vulnerable. Public Health.

[17] (2017). Thematic Report: Household Income Distribution in Hong Kong. Census and Statistics Department: Hong Kong Special Administrative Region.

[18] (2020). Thematic Household Survey Report No. 69. Census and Statistics Department: Hong Kong Special Administrative Region.

[19] Kwok KO, Li KK, Chan HHH, Yi YY, Tang A, Wei WI, Wong SYS (2020). Community Responses during Early Phase of COVID-19 Epidemic, Hong Kong. Emerg Infect Dis.

[20] Wang MP, Viswanath K, Lam TH, Wang X, Chan SS (2013). Social determinants of health information seeking among Chinese adults in Hong Kong. PLoS One.

[21] Li J, Zheng H (2020). Online InformationSeeking and Disease Prevention Intent During COVID-19 Outbreak. Journal Mass Commun Q.

[22] Luk TT, Zhao S, Weng X, Wong JY, Wu YS, Ho SY, Lam TH, Wang MP (2020). Exposure to health misinformation about COVID-19 and increased tobacco and alcohol use: a population-based survey in Hong Kong. Tob Control.

[23] Zhao SZ, Wong JYH, Wu Y, Choi EPH, Wang MP, Lam TH (2020). Social Distancing Compliance under COVID-19 Pandemic and Mental Health Impacts: A Population-Based Study. Int J Environ Res Public Health.

[24] Mitsutake S, Shibata A, Ishii K, Oka K (2016). Associations of eHealth Literacy With Health Behavior Among Adult Internet Users. J Med Internet Res.

[25] Shuai JG, Xiao MY, Yu YS, Dan N, Xue ML, Lu W (2013). Adaptation and evaluation of Chinese version of eHEALS and its usage among senior high school students. Chin J Health Educ.

[26] Hu L, Bentler PM (1999). Cutoff criteria for fit indexes in covariance structure analysis: Conventional criteria versus new alternatives. Struct Equ Modeling.

[27] Richtering SS, Hyun K, Neubeck L, Coorey G, Chalmers J, Usherwood T, Peiris D, Chow CK, Redfern J (2017). eHealth Literacy: Predictors in a Population With Moderate-to-High Cardiovascular Risk. JMIR Hum Factors.

[28] (2020). Infection prevention and control during health care when novel coronavirus (nCoV) infection is suspected: interim guidance, 25 January 2020. World Health Organization.

[29] (2019). Hong Kong Poverty Situation Report 2018. Government of the Hong Kong Special Administrative Region.

[30] (2017). Health Equity Assessment Toolkit (HEAT): Software for exploring and comparing health inequalities in countries. Built-in database edition. Version 2.0. World Health Organization.

[31] Din HN, McDaniels-Davidson C, Nodora J, Madanat H (2019). Profiles of a Health Information-Seeking Population and the Current Digital Divide: Cross-Sectional Analysis of the 2015-2016 California Health Interview Survey. J Med Internet Res.

[32] Zhao X, Fan J, Basnyat I, Hu B (2020). Online Health Information Seeking Using “#COVID-19 Patient Seeking Help” on Weibo in Wuhan, China: Descriptive Study. J Med Internet Res.

[33] Czaja S, Boot W, Charness N, Rogers W (2019). Individual differences. Designing for Older Adults. 3rd edition.

[34] (2020). Usage of Information Technology and the Internet by Hong Kong Residents. Census and Statistics Department: The Government of the Hong Kong Special Administrative Region.

[35] Jones NM, Thompson RR, Dunkel Schetter C, Silver RC (2017). Distress and rumor exposure on social media during a campus lockdown. Proc Natl Acad Sci USA.

[36] Rikard RV, Thompson MS, McKinney J, Beauchamp A (2016). Examining health literacy disparities in the United States: a third look at the National Assessment of Adult Literacy (NAAL). BMC Public Health.

[37] Neter E, Brainin E, Baron-Epel O (2015). The dimensionality of health literacy and eHealth literacy. Eur Health Psychol.

[38] Jayasinghe R, Ranasinghe S, Jayarajah U, Seneviratne S (2020). Quality of online information for the general public on COVID-19. Patient Educ Couns.

[39] Viswanath K, Finnegan JR (2016). The Knowledge Gap Hypothesis: Twenty-Five Years Later. Annals of the International Communication Association.

[40] Cowling BJ, Ali ST, Ng TWY, Tsang TK, Li JCM, Fong MW, Liao Q, Kwan MY, Lee SL, Chiu SS, Wu JT, Wu P, Leung GM (2020). Impact assessment of non-pharmaceutical interventions against coronavirus disease 2019 and influenza in Hong Kong: an observational study. Lancet Public Health.

[41] Hsu W, Chiang C, Yang S (2014). The effect of individual factors on health behaviors among college students: the mediating effects of eHealth literacy. J Med Internet Res.

[42] Kim S, Son Y (2017). Relationships Between eHealth Literacy and Health Behaviors in Korean Adults. Comput Inform Nurs.

[43] Chong YY, Cheng HY, Chan HYL, Chien WT, Wong SYS (2020). COVID-19 pandemic, infodemic and the role of eHealth literacy. Int J Nurs Stud.

[44] Sentell T, Vamos S, Okan O (2020). Interdisciplinary Perspectives on Health Literacy Research Around the World: More Important Than Ever in a Time of COVID-19. Int J Environ Res Public Health.

[45] Neter E, Brainin E (2017). Perceived and Performed eHealth Literacy: Survey and Simulated Performance Test. JMIR Hum Factors.

[46] Norman C (2011). eHealth literacy 2.0: problems and opportunities with an evolving concept. J Med Internet Res.

[47] Griebel L, Enwald H, Gilstad H, Pohl A, Moreland J, Sedlmayr M (2018). eHealth literacy research-Quo vadis?. Inform Health Soc Care.

