# Supplementary material for: Socioeconomic Disparities in eHealth Literacy and Preventive Behaviors During the COVID-19 Pandemic in Hong Kong: Cross-sectional Study
Source: J Med Internet Res. 2021 Apr 14;23(4):e24577. doi: 10.2196/24577 (PMC8048711; doi:10.2196/24577)
Supplement: Multimedia Appendix 1 [file jmir_v23i4e24577_app1.pdf]

Multimedia Appendix 1. eHealth Literacy Scale [a]

Item	Mean (SD)
I know how to find helpful health resources on the Internet	3.30 (1.12)
I know how to use the Internet to answer my questions about health	3.25 (1.11)
I know what health resources are available on the Internet	3.32 (1.12)
I know where to find helpful health resources on the Internet	3.30 (1.12)
I know how to use the health information I find on the Internet to help me	3.29 (1.11)
I have the skills I need to evaluate the health resources I find on the Internet	3.19 (1.11)
I can tell high quality health resources from low quality health resources on the Internet	3.25 (1.10)
I feel confident in using information from the Internet to make health decisions	3.21 (1.08)

[a] The scale was self-reported according to participants' past last experience using the Internet for COVID-19 related information.
