# Supplementary material for: Socioeconomic Disparities in eHealth Literacy and Preventive Behaviors During the COVID-19 Pandemic in Hong Kong: Cross-sectional Study
Source: J Med Internet Res. 2021 Apr 14;23(4):e24577. doi: 10.2196/24577 (PMC8048711; doi:10.2196/24577)
Supplement: Multimedia Appendix 2 [file jmir_v23i4e24577_app2.pdf]

Multimedia Appendix 2. Unweighted prevalence of preventive behaviors by online COVID-19 information seeking (N=1501).

	Sought (%)	Non-sought (%)	P
**Wear surgical masks**			<0.001
Never	16 (1.5)	33 (7.2)	
Occasionally	35 (3.4)	17 (3.7)	
Sometimes	90 (8.7)	52 (11.3)	
Often	899 (86.4)	359 (77.9)	
**Wear fabric masks**			0.11
Never	529 (50.9)	251 (54.5)	
Occasionally	192 (18.5)	73 (15.8)	
Sometimes	153 (14.7)	52 (11.3)	
Often	166 (16.0)	85 (18.4)	
**Washing hands with alcohol-based sanitizer**			<0.001
Never	9 (0.9)	17 (3.7)	
Occasionally	83 (8.0)	64 (13.9)	
Sometimes	376 (36.2)	189 (41.0)	
Often	572 (55.0)	191 (41.4)	
**Adding water/bleach to the household drainage system**			0.001
Never	42 (4.0)	25 (5.4)	
Occasionally	186 (17.9)	93 (20.2)	
Sometimes	427 (41.1)	221 (47.9)	
Often	385 (37.0)	122 (26.5)	
**Keeping a social distance from people in public areas (e.g., 1.5 meters)**			0.002
Never	34 (3.3)	18 (3.9)	
Occasionally	175 (16.8)	80 (17.4)	
Sometimes	454 (43.7)	241 (52.3)	
Often	377 (36.3)	122 (26.5)	
